# News and Miscellany

**Published:** 1903-11

**Authors:** 


### News and Miscellany.

LINEN UNDERWEAR is much more comfortable and hygienic than flannel. Flannel will not absorb perspiration and the wearer has frequently a damp or wet skin, and, of course, easily catches cold. Underwear should be boiled to sterilize and cleanse it. Flannels will not bear boiling; they shrink every time they are washed until they become as thick as leather, and as impervious. The editor of this journal personally recommends the Deimel Linen Mesh Underwear. It is a luxury. Since discarding flannel and using the Deimel Linen his health has greatly improved. The famous Deimel linen undershirts and drawers may be ordered direct from 491 Broadway, or from Smith & Wilcox, Harrell & Klein or Scarbrough & Hicks, Austin.

THE EAST TEXAS MEDICO-CHIRURGICAL SOCIETY will hold its regular semi-annual meeting at Palestine November 19th and 20th, inst. This is one of the largest district societies in the State and numbers amongst its members some of the ablest men in the profession. It is a delight to attend its meetings, but "Dan'els" has to cut it out this trip. Too many drafts for delinquent subscriptions coming back unpaid.

THE yellow fever mortality at Laredo to date (November 12th) has been about 10 per cent—789 cases and 78 deaths; at San Antonio, 18 cases reported and 7 deaths—about 40 per cent. At Linares and Monterey, Mexico, it is said to have been about 50 per cent. How is this difference to be accounted for?

THE JOURNAL'S friend, Dr. J. F. Eaves, of Millican, Texas, demonstrated before the Brazos Valley Medical Association, at Hearne, this week a new operation for intestinal anastomosis and resection. It was my misfortune to not be present. The Brazos Valley Medical Society embraces a large number of very excellent and progressive men, and they are up to date, you bet. I hope to publish Dr. Eaves' paper.

THE new pathologist of the Medical Department of the University of Texas, who succeeds Prof. Allen J. Smith, is Dr. Alfred E. Thayer. He is a graduate of the Medical Department of Columbia University, Class of 1884, and took special courses in pathology under the famous teachers in Gottingen and Vienna. He was at one time professor of pathology in the University of Virginia. At the time of his election to the chair of pathology in the Texas Medical College he was instructor in pathology and bacteriology at Cornell.

---

MISS LUCILE GARWOOD, the bright young daughter of Dr. A. Garwood, of New Braunfels, recently read before a local literary club a paper on "What I Know of Detention Camps, Disinfection and Mosquitoes," which was much applauded. It was said to be a "hit," and gives promise of real literary talent. Miss Lucile had been to San Antonio and the quarantine caught her.

---

THE EPILEPTIC ASYLUM, at Abilene, will not be ready for occupancy till January 1st, when it will be filled to its capacity—200. There are already over 600 applicants. Dr. Jno. Preston is superintendent.

---

SHE: "And would you still wish to marry me if I had an artificial optic?"

HE: "Yes, darling; with all thy false eye'd love thee still."—*Life.*

---

THE TOY PISTOL MUST GO.—At the twenty-ninth annual session of the Mississippi Valley Medical Association, held at Memphis, October 7th to 9th, the following resolutions were adopted:

In view of the fact that more than 400 deaths from tetanus occurred following the Fourth of July celebration of 1903, as shown by the statistical report elaborated by Dr. S. C. Stanton, of Chicago, and published in the *Journal of the American Medical Association* of August 29, 1903, the great majority of which might have been prevented had proper precautions been taken; therefore, be it

*Resolved,* That the conclusions which follow, as offered by Dr. Stanton in a paper presented before the Association, at the above meeting, be endorsed as the sense of the Association; and be it further

*Resolved,* That the Secretary be instructed to forward a copy of these resolutions and conclusions to the medical press, associated press, and the secretaries of the several State medical societies, with the request that they publish same and take suitable action thereon.

1. Enforcement of existing laws regarding the sale of toy pistols and other dangerous toys.

2. Enactment of laws by the nation, States and municipalities prohibiting the manufacture and sale of toy pistols, blank cartridges, dynamite canes and caps, cannon crackers, etc.

3. Open treatment of all wounds, however insignificant, in which from the nature or environment there is any risk of tetanus.

4. Immediate use of tetanus antitoxin in all cases of Fourth of July wounds, or wounds received in barnyards, gardens, or other places where tetanus infection is likely to occur.

5. As a forlorn hope, the injection of tetanus antitoxin after tetanus symptoms have appeared.

The Texas State Medical Association should take action on this important question.

---

ALKALOIDS AND THINGS.—We have received from the Abbott Alkaloidal Co. some of their elegant effervescent "Saline Laxative." It is splendid where a cooling aperient is needed. We have also received their little booklet "Alkaloidal Suggestions." You can get either or both by asking for it and mentioning the "Red-Back." I am indebted to them also for a neat little nine-vial morocco pocket-case of their alkaloids. This is rational medication.

---

THE VON BOECKMANN-JONES CO. are the best and most artistic printers in Texas. I "point with pride" to my October number as a specimen of neat, clean and high class typography. They take orders for all kinds of printing and binding, and can discount St. Louis or Chicago prices. Try 'em.

---

"I WANT some consecrated lye," he slowly announced as he entered the drug store.

"You mean concentrated lye?" suggested the druggist, as he repressed a smile.

"Well, maybe I do. It does nut-meg any difference. It's what I camphor, anyway. What does it sulphur?"

"A quarter a can."

"Then you can give me a can."

"I never cinnamon who thought himself so witty as you do," said the druggist in a gingerly manner, feeling called upon to do a little punning himself.

"Well, that's not bad ether," laughed the customer, with a syruptitious glance. "I ammonia novice at the business, though I've soda good many puns that other punsters get the credit of. However, I don't care a copperas far as I am concerned, though they ought to be handled without cloves till they wouldn't know what was the matter with them. Perhaps I shouldn't myrrh myrrh. We have had a pleasant time, and I shall caraway—"

It was too much for the druggist. He collapsed.—*American Druggist.*

---

Not Deserving of Recognition.—"By the way," said the gentlemanly looking person in the black broadcloth suit, "if you mention my name in connection with the accident, you may say that 'Dr. Swankem was called and the fractured arm was suitably bandaged,' or something to that effect. Please spell the name correctly. Here's my card."

"Thanks," said the reporter, looking at the card. "You are next door to Dr. Rybold, I believe. Are you acquainted with him?"

"No, sir," replied Dr. Swankem, stiffly. "We do not recognize Dr. Rybold as a member of the profession. He advertises."—*Chicago Tribune.*

---

Will Repair Your Electrical Machines.—Static and all electrical medical apparatus put in running order. I am also agent for electrical and X-ray apparatus. Oliver Brush, 710 Colorado Street, Austin, Texas.

---

New Orleans Polyclinic.—Seventeenth annual session opens November 2, 1903, and closes May 28, 1904. Physicians will find the Polyclinic an excellent means for posting themselves upon modern progress in all branches of medicine and surgery. The specialties are fully taught, including laboratory work. For further information, address New Orleans Polyclinic, postoffice box 797, New Orleans, La.

